# Privacy-Preserving Non-Wearable Occupancy Monitoring System Exploiting Wi-Fi Imaging for Next-Generation Body Centric Communication

**DOI:** 10.3390/mi11040379

**Published:** 2020-04-03

**Authors:** Syed Aziz Shah, Jawad Ahmad, Ahsen Tahir, Fawad Ahmed, Gordon Russell, Syed Yaseen Shah, William J. Buchanan, Qammer H. Abbasi

**Affiliations:** 1 School of Computing and Mathematics, Manchester Metropolitan University, Manchester M13 9PL, UK; s.shah@mmu.ac.uk; 2 School of Computing, Edinburgh Napier University, Edinburgh EH10 5DT, UK; g.russell@napier.ac.uk (G.R.); B.Buchanan@napier.ac.uk (W.J.B.); 3 Department of Electrical Engineering, University of Engineering and Technology, Lahore, Punjab 54890, Pakistan; ahsenkn001@gmail.com; 4 Department of Electrical Engineering, HITEC University Taxila, Punjab 47080, Pakistan; fawad@hitecuni.edu.pk; 5 School of Computing, Engineering and Built Environment, Glasgow Caledonian University, Glasgow G4 0BA, UK; yasinshah@gmail.com; 6 School of Engineering, University of Glasgow, Glasgow G12 8QQ, UK; Qammer.Abbasi@glasgow.ac.uk

**Keywords:** Wi-Fi, privacy, occupancy, deep learning, encryption

## Abstract

Nano-scaled structures, wireless sensing, wearable devices, and wireless communications systems are anticipated to support the development of new next-generation technologies in the near future. Exponential rise in future Radio-Frequency (RF) sensing systems have demonstrated its applications in areas such as wearable consumer electronics, remote healthcare monitoring, wireless implants, and smart buildings. In this paper, we propose a novel, non-wearable, device-free, privacy-preserving Wi-Fi imaging-based occupancy detection system for future smart buildings. The proposed system is developed using off-the-shelf non-wearable devices such as Wi-Fi router, network interface card, and an omnidirectional antenna for future body centric communication. The core idea is to detect presence of person along its activities of daily living without deploying a device on person’s body. The Wi-Fi signals received using non-wearable devices are converted into time–frequency scalograms. The occupancy is detected by classifying the scalogram images using an auto-encoder neural network. In addition to occupancy detection, the deep neural network also identifies the activity performed by the occupant. Moreover, a novel encryption algorithm using Chirikov and Intertwining map-based is also proposed to encrypt the scalogram images. This feature enables secure storage of scalogram images in a database for future analysis. The classification accuracy of the proposed scheme is 91.1%.

## 1. Introduction

Nano-scaled structures, wireless sensing, wearable devices, and wireless communications systems are anticipated to support the development of new next-generation technologies in near future. The exponential rise in future Radio-Frequency (RF) sensing systems has demonstrated its applications in areas such as wearable consumer electronics, remote healthcare monitoring, wireless implants, and smart buildings.The advances of low-cost small electronic devices, wireless sensing systems, real-time monitoring, and the Internet of Things (IoT) has played the role of a catalyst that has primarily expanded the horizon of these technologies. In this context, we introduce a privacy-preserving, device-free occupancy real-time occupancy monitoring system for energy optimizing in smart buildings.

The smart built environment essentially refers to the man-made environment providing the setting for different human activities including smart cities, large scale buildings, and beyond. It consumes a significant amount of electricity around the world and keeps on increasing day by day [1]. In this context, effective strategies are required to be developed in order to decrease the overall energy consumption while maintaining and improving the thermal comfort of occupants residing in buildings. Several studies indicate that smart lighting systems and Heating Ventilating and Air Condition (HVAC) save energy up to 30%, provided they work based on the suggestions given in [2]. The HVAC system can automatically turn off in unoccupied settings and the ventilation rate can be adjusted as well depending on the total number of occupants present to optimize the energy consumption. In addition to energy-saving, the adaptive crowd density control scheme can be applied by estimating occupant number in various places.

For example, optimized services provided at shopping malls, hotels, restaurants, and transportation stations can be allotted to improve customer care service. Crowd density control and counting can enhance the indoor evacuation process in emergency cases. Hence, the design of a low-cost, robust, accurate, safe, and secure occupancy monitoring system is of utmost importance that can count the number of occupants while keeping the privacy preserved. Presently, the majority of the occupancy monitoring and detection systems use infrared sensors. However, these systems present a huge number of false alarms when occupants are moving slowly. Furthermore, state-of-the-art occupancy detectors estimate the total number of people in the indoor environment as well. Camera-based systems [3] are one of the most used detectors. However, the person has to be in line-of-sight with sufficient light; dedicated cameras have to be deployed; and it raises privacy concerns as well. Several researchers have used Radio Frequency (RF) such as Bluetooth [4], Radio Frequency Identification (RF) [5], and sensor fusion [6] to detect occupants. The limitation of the aforementioned RF technologies is that a person has to carry RF or Bluetooth tags all the time within the area of interest. In addition to the limitation of RF sensing devices, some of the main advantages are that it is inexpensive, robust, and accurate with no issue of privacy. The ubiquitous Wi-Fi routers have been widely used for various application, ranging from remote health monitoring to fall detection, and so on [6,7,8,9]. The Wi-Fi technology is the best possible solution for occupancy monitoring in an indoor setting as it transmits the signal in omnidirections, covering almost the entire indoor area.

The Received Signal Strength Indicator (RSSI) obtained from Wi-Fi signals has been used to estimate occupants. The disadvantage of RSSI is that it suffers from coarse grain resolution, is highly susceptible to noise, and presents fluctuations, making it an infeasible solution for occupancy detection. On the other hand, channel state information extracted from Wi-Fi signals provide multiple frequency sub-channels, where one or more frequency carriers can be used to detect a person in an indoor environment. In this paper, we propose a novel low-cost, easily deployable, device-free, privacy-preserving Wi-Fi technology-based occupancy detection system using commercially available, off-the-shelf wireless devices such as Wi-Fi router, network interface card. and an omnidirectional antenna. In this research, two independent modules were implemented: (i) deep learning for occupancy detection; and (ii) chaos-based scalogram image encryption.

## 2. Related Work

Numerous researchers have introduced different RF sensing-based occupancy monitoring systems over the past few years [10,11,12,13,14,15]. In this regards, this section introduces some of the most commonly used RF sensing techniques to estimate the occupant within the area of interest, along with their advantages and limitations. For instance, the authors of [16] used Passive Infrared Sensor (PIR) to detect a person within the specific zone. The system identifies the presence of a person by estimating the variances in radiation emitted from the source (sensor in this case). Furthermore, Duarte et al. [17] exploited the PIR device to measure occupancy in real-time at various zones, comprising of different rooms. Occupancy detection system using two PIR sensors were used in [18,19] to localize the occupant. The main advantage of this technology is its low cost and low power consumption level. However, the PIR sensor fails to detect intricate or stationary occupants due to its limited range resolution. Camera-based technology for occupancy monitoring is another method for crowd estimation and person monitoring. This system uses frames extracted from video recordings, where accurate and precise information about the occupant can be obtained. The image processing classification technique has three main essential steps: background score subtraction from the main body, movement detection, and occupant identification. The limitation of the vision-based technique is that it is dependent on the light intensity and the occupant has to be in line-of-sight of the camera, which raises privacy concerns as well. On the other hand, some researchers have used the Bluetooth Low Energy (BLE) module for crowd counting and occupancy estimation [4]. Deploying multiple iBeacons in the area of interest (indoor setting), the occupant can be estimated using RSSI in combination with machine learning algorithms such as support vector machine, K-nearest neighbor, etc.. The minimum requirement in the deployment of the BLE module is its biggest limitation to being implemented. To the best of the authors’ knowledge, none of the existing monitoring systems consider the cost, easy deployment, security, and privacy preservation aspects. This paper addresses all the open areas that have not been addressed so far.

## 3. Security and Privacy in Body Centric Communication

Body centric communication systems need specific privacy and security protocol to ensure the privacy, confidentiality, and data integrity of a person’s information. A supporting body centric communication infrastructure should deploy particular security measures that ensure all aforementioned features [20]. A comprehensive survey of main challenges (security and privacy) in wireless body area networks is presented in detail in [21]. Preserving and securing privacy of important data from adversaries without modifications, digital model learning, and sharing private information in body centric communication are extremely challenging. In an ideal world, the privacy preservation require available datasets always be secured from outside the data by the users.The private privacy should be enabled throughout the communication process of a given task.However, fool-proof ideal privacy preservation is impossible. For example, deploying fully-secured image encryption techniques including an AES-256 encryption scheme that is used to secure the available information from potential threats can harm typical delivering services in body area network. On the contrary, agreeing with simple and straightforward encryption schemes, such as information anonymization, is ineffective against breaches. The selection of encryption schemes also plays an important role in the designing of preserving data for next-generation body centric communications. Data and information protection schemes make space operations that are limited on the obscure data, while complex data protection techniques must be separate from simple schemes. For instance, a homomorphic encryption system is introduced in [22] to secure information using data mining techniques in the context of plain homomorphic multiplications. In addition, privacy-preserving schemes have a rapid rise in computational costs, thus often making it infeasible to be deployed in real-world applications. For instance, the overall cost of an effective multiplication-based homomorphic encryption algorithm transforming a plaintext number into a 256-byte ciphertext with a 1024-bit security key within 5 s sums to 1500 s for such encrypted ciphertexts. Hence, it is very important to divide the collective workload of encryption schemes to corresponding distributors in relation to the available resources.

## 4. Wi-Fi Sensing for Occupancy Monitoring

**Figure 1:**
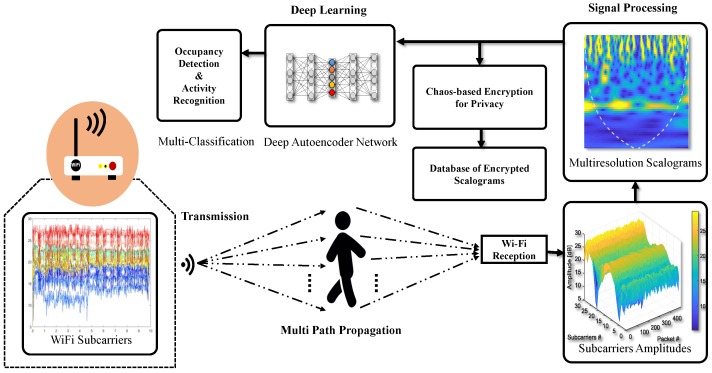
Architecture for an occupancy monitoring system based on Wi-Fi signals, driven by deep network.

The received signal strength indicator and the Channel State Information (CSI) extracted are two of the most widely used types of information, applicable in various areas of wireless communication. RSSI measurements only present simple signal strength in the context of signal propagation, which is why this information is inadequate for occupancy monitoring due to its unstable nature. Zou et al. [23] presented an occupancy monitoring system based on RSSI measurements that could obtain an overall accuracy of nearly 70%. On the contrary, the CSI measurements obtained using low-cost wireless devices provide fine-grained information by exploiting multiple frequency subcarriers [24]. The overall proposed architecture based on Wi-Fi signals is given Figure 1.

**Figure 2:**
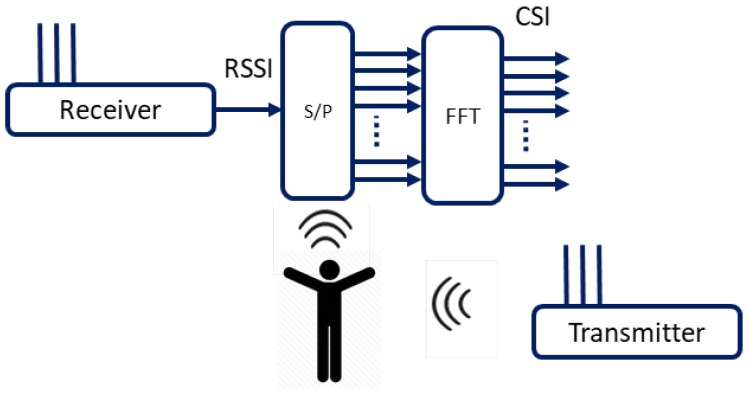
Frequency carrier conversion: time domain to frequency domain using RSSI/CSI.

The Wi-Fi technology powered by IEEE 802.11 a/a/ac utilizes Orthogonal Frequency Division Multiplexing (OFDM), effectively encountering the multipath propagation effect caused in indoor settings due to physical obstructions such as walls, ceiling, floor, etc. In OFDM, the frequency carrier is divided into multiple (orthogonal) subcarriers where the data stream [25,26]. The Wi-Fi signal received, i.e., the pass-band signal, is converted into message or baseband signal. The orthogonal frequency subcarriers are transformed into the frequency domain from the time domain by applying serial-to-parallel converter on RF signal, and Fast Fourier Transform (FFT) is then applied on all received subcarrier, as shown in Figure 2.

A low-cost, off-the-shelf small wireless device, such as Atheros ar5b225, can be used to extract the CSI information from OFDM subcarriers. Open-source network interface card wireless device drivers reveal the measured CSI for all subcarriers, providing fine-resolution wireless channel information, comprising wireless medium characteristics including multipath fading, reflection, refraction, and shadowing effect.

**Figure 3:**
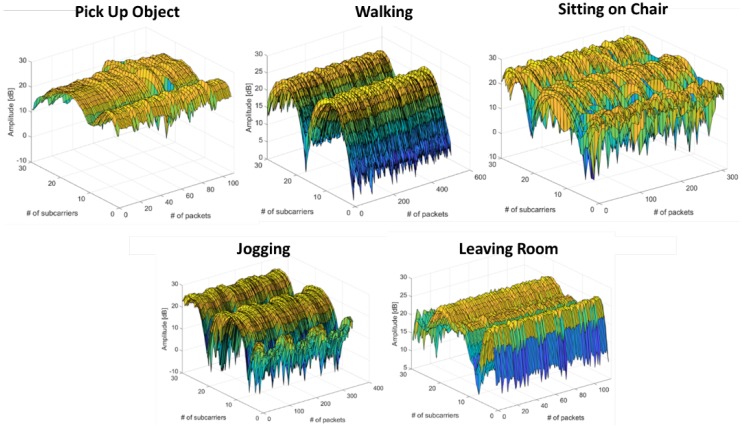
Variances of amplitude information against time and frequency domain.

Let $H_{i}$ represent the CSI data for *i*th subcarrier that carries complex information and is denoted as follows:

(1) $$H_{i}=∥H^{i}∥e^{j∠H_{n}}$$

where $|H_{i}|$ and $∠H_{i}$ provide the variances of amplitude and phase information for *i*th frequency channel, respectively. The phase information extracted from CSI data for single frequency channel $i,∠H_{i}$ is expressed as:

(2) $$H_{i}=∠H_{i}+\left(\lambda_{p}+\lambda_{s}\right)m_{i}+\lambda_{c}+\beta +Z,$$

where β is the phase offset for *i*th frequency subcarrier and mi is the frequency channel number. The internal noise of the network interface card and external noise is denoted as *Z*, and $\lambda_{p}$, $\lambda_{s}$, and $\lambda_{c}$ are the phase errors, sample subcarrier offset, and central frequency offset, respectively. The raw channel state information is sufficient to extract meaningful information for occupancy monitoring due to the random noise present in Wi-Fi signals. In this context, we only use variances of amplitude information extracted from CSI data. The raw CSI measurements for five different activities, including occupant leaving the room, are shown in Figure 3. The proposed system deals with interference produced by other devices working in the 2.4 GHz band (microwave ovens, alarms, remote controls, Wi-Fi networks used for communications, etc.). The technical reasons are discussed as follows:

(1) Wi-Fi router is deployed closer to the receiving antenna.

(2) We avoid using other wireless systems nearby common sources of interference, such as power cables, microwave ovens, fluorescent lights, and cordless phones.

(3) We minimize the total number of live devices using the same RF band.

(4) Deploying the Wi-Fi router close to the receiving antenna enables the receiver to get the maximum power, thus other nearby devices operating on same frequency do not affect the proposed system.

### 4.1. Data Processing and Signal Acquisition

The channel station information recorded using the network interface card connected with the Wi-Fi router is discussed in this section. The CSI data are recorded from the data stream obtained from the Internet Control Messages Protocol (ICMP) data. In principle, the overall recorded CSI data are the same when compared to the ICMP data stream. Nonetheless, it was noticed that marginally fewer CSI data packets were transmitted than ICMP packets. To synchronize the frequency of the recorded data packets, we applied linear transformation function on CSI data recorded in raw form. In theory, the OFDM subcarriers that Wi-Fi signal exploit should carry independent data. However, in practice, the neighboring frequency subchannels carry similar information most of the time. To detect occupancy and obtain separate information from each subcarrier, we applied principal component analysis (PCA) to get independent datasets for each observation. The CSI data stream can be easily incorporated into several independent principle components.

### 4.2. Experimental Setup and Trials

**Figure 4:**
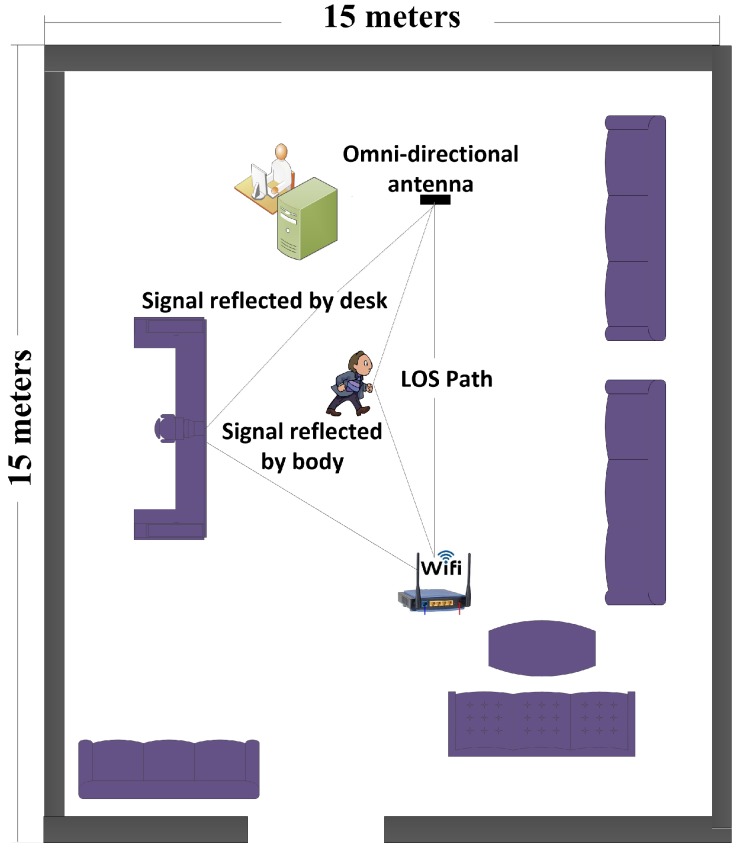
Experimental setup and trials for occupancy monitoring.

We conducted extensive experiments and trials in a hall, as described in Figure 4. The experiment was conducted on 15 subjects who were asked to do five ADLs. The age range of all subjects was 20–60 years. The transmitter (Wi-Fi router) and receiving antenna were placed 5 m away from each other at height of 1 m. The firmware introduced in [7] was used to record raw CSI and was installed in a desktop PC that continuously received OFDM packets. To identify occupants in indoor settings, we performed five sets of activities, as described above. Each human body movement brings a unique change in wireless channel that was inferred to identify activities performed by occupant in indoor environment.

### 4.3. Scalogram for Activity Detection and Occupancy Estimation

The multiresolution scalograms, represented in terms of time–frequency, obtained from channel state information packets were used to estimate the occupancy within indoor settings. The scalograms are energy density function retrieved by applying Continuous Wavelet Transform (CWT) on 1000 CSI data packets. The energy density function E(t,f) can be obtained from variances of the amplitude information of the CWT function $C_{d}\left(t,f\right)$ by applying a squaring function on a discrete sequence. The time–frequency measurement can be calculated from the CWT $C_{c}\left(t,s\right)$ of a Wi-Fi signal x(t), denoted as time *t* and scale *s*, as described in Equation (3).

(3) $$C_{c}\left(t,s\right)=\int_{-\infty }^{+\infty }x\left(v\right)\frac{1}{\sqrt{s}}\psi \left(\frac{v-t}{s}\right)dv$$

where $\psi (\frac{v-t}{s})$ is the dilation of the wavelet ψ(t). The expression v−t=τ is scaled down to the value of s as denoted as a function of frequency f, given $s=g_{1}\left(w\right)=g_{2}\left(f\right)$. The continuous wavelet transform of Wi-Fi signal for CSI data packets are expressed in Equation (4):

(4) $$C_{c}\left(t,f\right)=\int_{-\infty }^{+\infty }x\left(t+kT\right)\psi \left(kT,f\right)d\tau$$

where x(KT) is a discrete sequence of samples with a time period T=1/F, and the value of F represents the sampling frequency of an RF signal. The continuous wavelet transform of a discrete Wi-Fi signal can be acquired when the expression x(kT) is substituted with $CSI^{SC}\left(kT\right)$, written as:

(5) $$C_{d}\left(t,f\right)=T\sum_{k}CSI^{SC}x\left(t+kT\right)\psi \left(kT,f\right)d\tau$$

The value of *f* in Equation (6) was set to 60 Hz, i.e., the sampling frequency of variance of amplitude information of Wi-Fi signals, and the value of *T* was set to 03 ms. In this study, we chose mother wavelet, which is also known as the “morse” wavelet. The scalogram E(t,f) of the Wi-Fi signals can be elaborated further as:

(6) $$E=C_{d}\left(t,f\right)\times C_{d}^{*}\left(t,f\right)$$

(7) $$E=T^{2}\sum_{k1}\sum_{k2}CSI^{SC}x\left(t+k_{1}T\right)CSI^{SC^{*}}x\left(t+k_{2}T\right)\psi \left(k_{1}T,f\right)\psi^{*}\left(k_{2}T,f\right)$$

**Figure 5:**
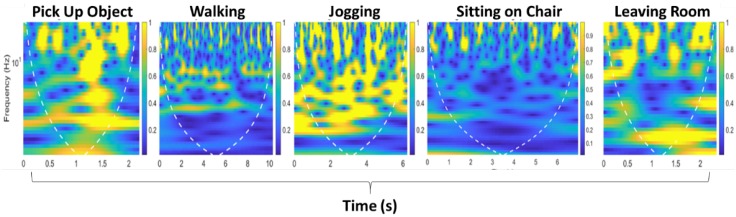
Scalograms obtained from variances of amplitude information when occupant for present in area of interest.

The CWT-based time–frequency scalograms provided fine-grained resolution analysis, independent of time window size. Consequently, they result in different dilations of morse wavelet. The time–frequency scalograms give significant variations in terms of CSI amplitude information due to the presence of an occupant within the indoor environment and give granular resolution due to small window time size at higher frequencies. Furthermore, the scalograms extracted have the potential to identify intricate features in RF signals due to large time window durations at lower RF frequencies. The scalograms shown in Figure 5 are produced against the frequency domain (logarithmic scale). The white dotted line represents the cone of influence that splits the region where edge effects are significant to identify occupant presence within the indoor environment.

### 4.4. Autoencoder for Scalogram Classification

**Figure 6:**
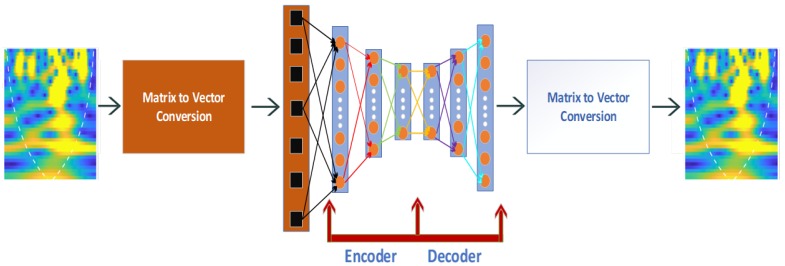
Deep autoencoder-based classification.

The biggest challenge that researchers face is the classification of RF signals due to the limited amount of data as a huge amount of time is required for data collection. To cope with the small number of observations, we used the autoencoder neural network, which delivers the best classification performance when exposed in such scenarios [27,28,29]. The autoencoder classifier provided the input data at the output, as shown in Figure 6. For example, for input value x, the neural network tries to find a function, namely, hw(x)≈x. The unsupervised algorithm was introduced to initialize the weights and biases of an autoencoder, which was extremely effective when limited training data were available. This algorithm implements unsupervised data processing (pre-training) by encoding and decoding the available datasets, respectively. It also estimates a nonlinear mapping on given datasets as an input *x* that is expressed as follows.

(8) $$z_{i}=\sigma \left(\hat{W}e_{i}+\tilde{b}\right)$$

where $\hat{W}$ and $\tilde{b}$ are weights and biases, respectively. The autoencoder classifier tries to reduce the error rate by minimizing the following values:

(9) $$J\left(\theta \right)=\frac{1}{N}\sum_{i=1}^{N}{\left(x_{i}-z_{i}\right)}^{2}$$

To optimize the autoencoder neural network, cost function in conjunction with a sparsity parameter is implemented to drive the neural network for learning the correlation when different inputs are given [30]. In addition to the these parameters, the cost function can be expressed as follows:

(10) $$gmin_{\left(\theta \right)}J\left(\theta \right)=\frac{1}{N}\sum_{i=1}^{N}{\left(x_{i}-z_{i}\right)}^{2}+\beta \sum_{i=1}^{N}KL(p||p_{l})$$

where *h* denotes the number of hidden neurons, β is the sparsity proportion, and KL describes Kullback–Leibler divergence and can be expressed as follows:

(11) $$KL(p||p_{l})=p\log\left(\frac{p}{p_{j}}\right)+\left(1-p\right)\log\left(\frac{1-p}{1-p_{j}}\right)$$

### 4.5. The Proposed Encryption Scheme

**Figure 7:**
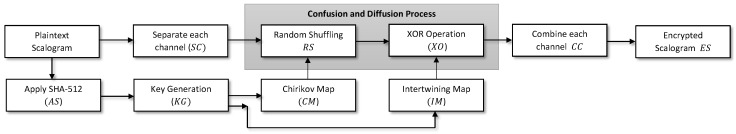
Flowchart of the encryption process.

**Figure 8:**
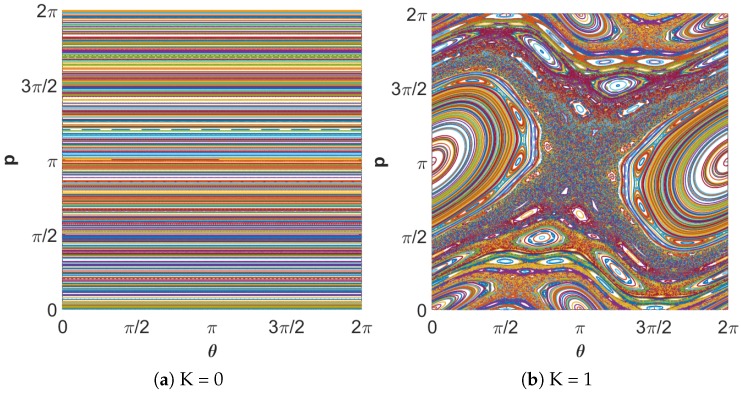

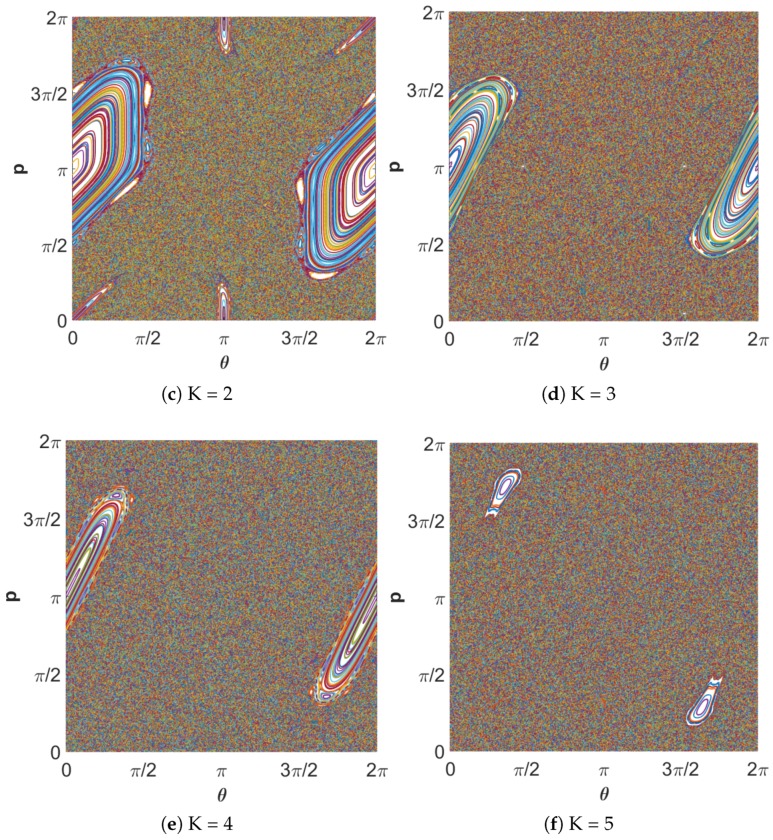
Chaotic orbits of the standard map for different values of *K*.

The scalogram images are encrypted with lightweight Chirikov and Intertwining maps. The flowchart of the encryption process is highlighted in Figure 7. One can see in Figure 7 that both confusion and diffusion steps are deployed for protecting the privacy from eavesdroppers. Due to lightweight nature, pseudo-randomness, ergodicity, and dynamic behavior, the chaos-based algorithm is used in this work. Two chaotic maps, Chirikov standard map and Intertwining map, are used during the encryption. Mathematically, Chirikov standard map is written as:

(12) $$\begin{array}{c} \left\{\begin{array}{cc} \alpha_{n+1} & =\alpha_{n}+K\sin\theta n\;\mathrm{mod}\;2\pi \\ \theta n+1 & =\theta_{n}+\alpha_{n+1}\;\;\mathrm{mod}\;2\pi \end{array}\right. \end{array}$$

where *K* is control parameter, and $\alpha_{n}$ and $\theta_{n}$ are real values between (0,2π). The constant coefficient *K* influences the degree of chaos exhibited by the map, as highlighted in Figure 8.

**Figure 9:**
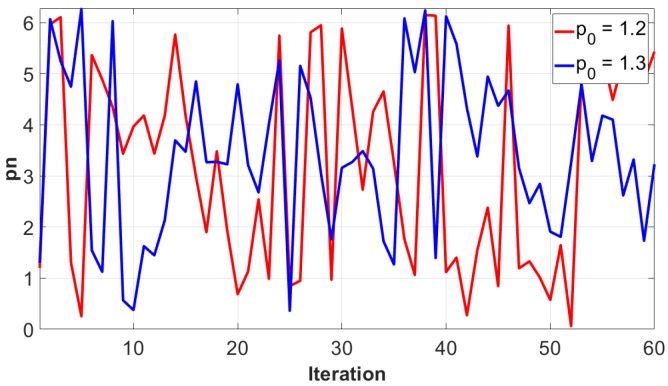
Output of Chirikov standard map for slightly different values of initial *p*.

Each plot is on the θα-plane, and it can be seen that increasing the value of *K* leads to a rich set of dynamics. It is obvious in Figure 8a that the map has regular values for *K* = 0. The chaotic region grows for higher values of *K*. In a chaotic cryptographic primitive, θ and *p* represent hidden inputs [31], with the other parameters akin to the group setting in Diffie–Hellman, the RSA prime, or the curve used in elliptic curve cryptography. The secret parameters are protected by the difficulty of determining $\left(\theta_{0},\alpha_{0},K\right)$ given $\left(\theta_{i},\alpha_{i},K\right)$, after the chaotic map has been iterated *i* times. For a secure crypto-system, the keyspace should be more than $2^{100}$. If the computational precision is $10^{-14}$, the keyspace is $\approx 2^{140}$, which indicates that the output of the Chirikov standard map is secure against brute-force attack. Moreover, slightly different values of α cause different output, which highlights the key sensitivity of the chaotic map, as shown in Figure 9. The key sensitivity test illustrates the strength of the Chirikov standard map.

During the diffusion step, the intertwining map is used, which is written as:

(13) $$\begin{array}{c} x_{n+1}=\left(\lambda \times \alpha \times y_{n}\times \left(1-x_{n}\right)+z_{n}\right)mod\left(1\right),\\ {\;y}_{n+1}=\left(\lambda \times \beta \times y_{n}+z_{n}\times \frac{1}{1+{\left(x_{n+1}\right)}^{2}}\right)mod\left(1\right),\\ {\;z}_{n+1}=(\lambda \times \left(x_{n+1}+y_{n+1}+\gamma \right)\times sin\left(z_{n}\right)mod\left(1\right). \end{array}$$

where $x_{n}$, $y_{n}$, and $z_{n}\in$ (0,1), 0≤λ≤3.999, |α|>33.5,|β|>37.9,|γ|>35.7.

**Encryption Steps**:

1. Let *I* be the original image scalogram having size m×n. Apply SHA-512 to get a hash value for initial conditions that can be utilized in chaos maps.
2. Save SHA-512 results in ω. The hexadecimal value is ω = $\omega_{1}\omega_{2}\;\ldots \;\omega_{128}$ = H1H2…H128, where $H1=2^{0}\times 2^{1}\times 2^{2},\;\ldots 2^{15}$.
3. Generate keys from the hash value. Convert hash value to decimal and apply modulus operation and set initial conditions for Chirikov standard map:
  $\alpha_{i}$ = convert2decimal($\omega_{1}\omega_{2}\ldots \omega_{32}$).
  $\alpha_{0}$ = $\frac{\alpha_{i}}{\zeta }$ mod(2π), where ζ is $3.40\times 10^{38}$.
  $\theta_{i}$ = convert2decimal($\omega_{33}\omega_{34}\ldots \omega_{64}$).
  $\theta_{0}$ = $\frac{\theta_{i}}{\zeta }$ mod(2π).
4. Set initial condition value for Intertwining map:
  $x_{i}$ = convert2decimal($\omega_{65}\omega_{66}\ldots \omega_{96}$).
  $x_{0}$ = $\frac{x_{i}}{\zeta }$ mod(1).
  $y_{i}$ = convert2decimal($\omega_{97}\omega_{3}4\ldots \omega_{128}$).
  $y_{0}$ = $\frac{y_{i}}{\zeta }$ mod(1).
  $z_{0}$ = ($x_{0}+y_{0}$) mod(1).
5. Separate each red, green, and blue channel and save the results in ψ, δ, and η, respectively.
6. Iterate Chirikov map 3×m×n times, randomly shuffle each pixel of ψ, δ, and η, and the save results in $\psi_{p}$, $\delta_{p}$, and $\eta_{p}$, respectively, through the random sequences obtained from Chirikov map.
7. Iterate Intertwining map 3×m×n times, multiply the obtained value with $10^{14}$, and save the results in a row matrix *A*. Apply the modulus operator and save the results in *B*:
  *B* = A mod(256).
8. Reshape *B* into three separate matrices, i.e, $B_{1}$, $B_{2}$, and $B_{2}$, and apply XOR operation:
  $C_{1}=\psi_{p}⊕$$B_{1}$.
  $C_{2}=\delta_{p}⊕$$B_{2}$.
  $C_{3}=\eta_{p}⊕$$B_{3}$.
9. Slightly change the initial conditions by adding a value σ=0.001:
  $\alpha_{0}$ = ($\alpha_{0}$ + σ) mod (2π).
  $\theta_{0}$ = ($\theta_{0}$ + σ) mod (2π).
  $x_{0}$ = ($x_{0}$ + σ) mod(1).
  $y_{0}$ = ($y_{0}$ + σ) mod(1).
  $z_{0}$ = ($x_{0}$ + $y_{0}$ + σ) mod(1).
10. Repeat Steps 6–9 ϵ times, and select ϵ = 4 for a good confusion and diffusion.
11. Combine each channel, $C_{1}$, $C_{2}$, and $C_{3}$, and save the encrypted scalogram results in *C*.

### 4.6. Encryption and Security Analysis

**Figure 10:**
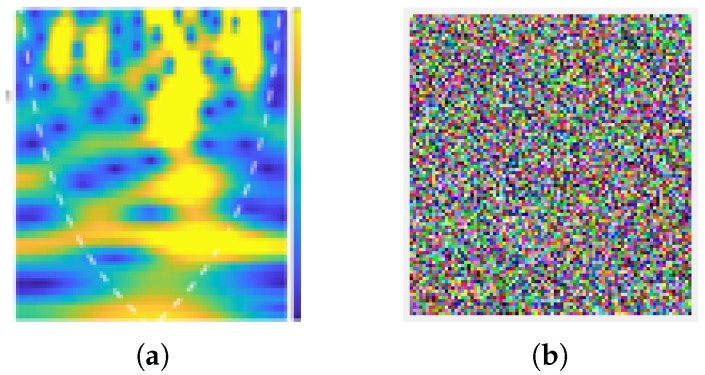
(**a**) Pick up original scalogram; and (**b**) encrypted scalogram.

**Figure 11:**
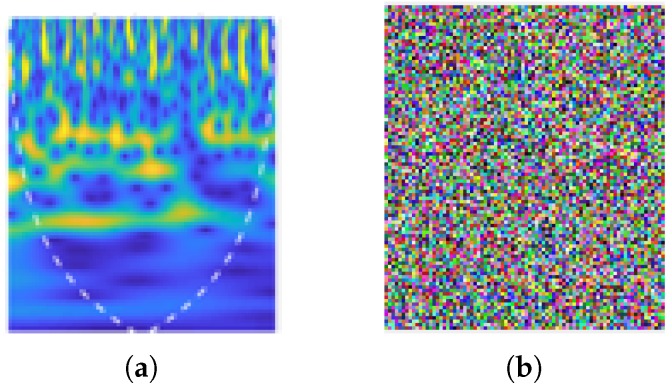
(**a**) Walking original scalogram; and (**b**) encrypted Scalogram.

The proposed scheme was tested on pick up object scalogram. The original and encrypted scalogram of pick up object are shown in Figure 10a,b, respectively. Additionally, encrypted walking scalogram is shown in Figure 11b. In Figure 10b and Figure 11b, it is clear that contents of scalogram are encrypted and an eavesdropper cannot predict the original activity. Visually, it is clear that contents are hidden; however, the security of an encryption scheme should be highlighted thorough a number of parameters, as outlined in our previous work [32,33]. Several parameters in Table 1 indicate the robustness and higher security of the proposed scheme. How such security parameters reflect robustness the proposed scheme can be found in [32,34,35,36].

## 5. Classification Results

We implemented the autoencoder model in a MATLAB tool, where training, validation, and testing were performed on scalograms generated from Wi-Fi signals. The neural network was trained for 200 epochs with a minibatch size of 90. The performance accuracy of the proposed system was obtained by dividing 20% of the training datasets as the validation set, and the model was evaluated after the completion of each iteration.

The adaptive moment estimation technique was used for optimizing the given datasets during the pre-training stage for a fine-tuning learning rate of 0.002. The grid search method was used during the process where optimized values for width and depth overcoming the overfitting problem are shown in Table 2. The three-layer unsupervised autoencoder had layer depths of 200, 100, and 50, respectively. The optimum classification performance in terms of percentage accuracy is 91.1%, as highlighted in Table 2.

## 6. Conclusions

This paper presents the application of next-generation body centric communication towards occupancy monitoring that can provide an effective and privacy preserved solution for reducing the energy consumption and carbon footprint. In the proposed model, non-wearable wireless devices such as Wi-Fi router, network interface card, and omnidirectional antennas operating at 2.4 GHz, are used to acquire data. Continuous wavelet transform is applied to the acquired RF signals to obtain Wi-Fi images that are processed using a deep learning algorithm to detect occupancy and to further perform occupancy classification. An unsupervised auto-encoder algorithm is used to classify images corresponding to different human activities of the occupants present in the area of interest. The performance of the proposed system was evaluated in terms of percentage accuracy, providing an overall accuracy of more than 91%. Lightweight image encryption techniques using multi-chaotic maps are proposed to encrypt the scalogram images. This feature enables secure storage of scalogram images for future usage such as improving training and testing accuracy of deep neural model.

## Figures and Tables

**Table 1:** Evaluation of the scheme through a number of security parameters.

Security Parameter	Original Pick up Scalogram	Encrypted Scalogram	Original Walking Scalogram	Encrypted Scalogram
CorrCoff (H)	0.9060	0.1599	0.8753	0.0644
CorrCoff (V)	0.9425	0.1245	0.9448	0.0341
CorrCoff (D)	0.8721	0.0900	0.8140	0.0068
Entropy	7.1273	7.7068	6.7482	7.9422
Key Sensitivity	NA	99.4311%	NA	99.6735%
NPCR	NA	99.4362 %	NA	99.6575%
UACI	NA	33.2151	NA	33.4512
Contrast	1.6186	10.0731	1.6889	10.5830
Homogeneity	0.8059	0.4228	0.7866	0.3944
Energy	0.1067	0.0197	0.1405	0.0162

**Table 2:** Optimized parameters for autoencoder (scalograms/Wi-Fi Sensing).

#	Width	Depth	Accuracy
1	20	1	77.3
2	50	1	78.1
3	100	2	76.7
4	50–100	2	80.0
5	150–200	3	88.0
**6**	**50-100-200**	**3**	**91.1**
7	10–25–50–100	4	81.3
8	15–30–60–200	4	80.7
9	30–60–120–240	5	81.5
10	40–80–240–300	5	79.9
11	15–30–45–90–200–400	6	80.9
12	50–100–200–400–800	6	85.5
